# Association between clustering of unhealthy lifestyle factors and risk of new-onset atrial fibrillation: a nationwide population-based study

**DOI:** 10.1038/s41598-020-75822-y

**Published:** 2020-11-05

**Authors:** So-Ryoung Lee, Eue-Keun Choi, Hyo-Jeong Ahn, Kyung-Do Han, Seil Oh, Gregory Y. H. Lip

**Affiliations:** 1 Department of Internal Medicine, Seoul National University Hospital, 101 Daehak-ro, Jongno-gu, Seoul, 03080 Republic of Korea; 2 Department of Internal Medicine, Seoul National University College of Medicine, Seoul, Republic of Korea; 3 Statistics and Actuarial Science, Soongsil University, Seoul, Republic of Korea; 4 Liverpool Centre for Cardiovascular Science, University of Liverpool and Liverpool Chest & Heart Hospital, Liverpool, UK; 5 Department of Clinical Medicine, Aalborg University, Aalborg, Denmark

**Keywords:** Cardiology, Risk factors

## Abstract

We aimed to investigate the association between the combination of unhealthy lifestyle and risk of AF. Subjects aged 66 years who underwent health examination from 2009 to 2015 were included. The cohort was divided into 8 groups by the combination of unhealthy lifestyle including current smoking, heavy drinking (> 30 g/day), and lack of regular exercise, and followed up for new-onset AF till December 31, 2017. Among 1,719,401 subjects, 47,334 had incident AF (5.5 per 1000 person-years) during a 5-year mean follow-up period. Lack of regular exercise was the most powerful factor to be associated with a higher risk of AF as a single factor (adjusted hazard ratio 1.11, 95% confidence interval 1.08–1.13). Amongst combinations of two unhealthy lifestyle factors, current smoking with heavy drinking, lack of regular exercise with heavy drinking, and lack of regular exercise with current smoking were associated with a 6%, 15%, and 20% higher risks of AF, respectively. A cluster of three unhealthy lifestyle components was associated with a 22% higher risk of AF. Increased numbers of unhealthy lifestyle factors were associated with a higher risk of incident AF. These findings support the promotion of a healthy lifestyle to lower the risk of new-onset AF.

## Introduction

Atrial fibrillation (AF) is the most common sustained cardiac arrhythmia encountered in clinical practice. The prevalence of AF is increasing globally, even in Asians with an ageing population and the adoption of a sedentary lifestyle^1–4^. AF is associated with increased risks of stroke, heart failure, dementia, and both cardiovascular and all-cause death; hence, the healthcare burden related to patients with AF has been increasing^4–6^.

Despite advances in management and treatment for AF, effective primary prevention strategies should be instituted to reduce the healthcare burden caused by new-onset AF. Several reports have elucidated the non-modifiable and modifiable risk factors for AF^7–15^. Framingham Heart Study reported the independent risk factors for incident AF included age, hypertension, diabetes mellitus, congestive heart failure, coronary heart disease, and valvular heart disease^16^. Beyond permanent or non-modifiable risk factors, more attention should perhaps be directed to modifiable factors, such as unhealthy lifestyle including lack of regular physical activity, smoking, and excessive alcohol consumption for prevention of incident AF.

The impact of each individual component of these unhealthy lifestyle factors on the incidence of AF has previously been reported^9,11,17,18^. Briefly, former or current smoking, heavy alcohol consumption, and habitual vigorous exercise were associated with increased risk of incident AF, and moderate physical activity was associated with reduced risk of AF^9,12,19–21^. However, these unhealthy lifestyle risk factors are infrequently seen in isolation, and are commonly clustered in the same individual leading to different effect sizes on the risk of clinical outcomes. Nevertheless, evidence is limited regarding the impact of the combination(s) of unhealthy lifestyle risk factors including low physical activity, smoking, and excessive alcohol consumption on incident AF^22–24^.

In this study, we aimed to investigate the association between the clustering of unhealthy lifestyle factors, defined by the combinations of smoking, alcohol consumption, and lack of physical activity and the risk of AF in a nationwide population cohort of subjects aged 66-year-old.

## Methods

### Data source and study population

We used the national health claims database linked with a health checkup database established by the Korean National Health Insurance Service (NHIS). The Korean NHIS is a single-insurer managed by the Korean government, and the entire Korean population (approximately 50 million) are mandatory subscribers^25,26^. The Korean NHIS offers universal and comprehensive medical care coverage for all enrollees. The Korean NHIS database contains all medical expenses claims data of the entire Korean population including subjects’ demographic information, diagnoses, examinations, prescription dispensing records, and procedure for inpatient and outpatient services. Diagnoses were coded using the *International Classification of Disease, Tenth Revision, Clinical Modification* codes. In Korea, among the entire population, subjects aged 66 years old are strongly recommended to receive a standardized health checkup provided by the Korean National Health Insurance Corporation (NHIC), called “Transitional age health screening”^25^. The health checkup includes measurements of height, weight, waist circumference, and self-reporting questionnaires about health-related behaviors such as smoking status, alcohol consumption, and exercise habits. This study complied with the Declaration of Helsinki. Because of the inherent characteristics of the database, this study was exempted from review by the Institutional Review Board of the Seoul National University Hospital (E-1811-069-984) and the requirement for informed consent was waived.

Using this database for the population from 2009 to 2015, we included subjects aged 66 years who were indicated for a health examination for ‘life transitions period.’ Namely, all study patients were 66 years old at the baseline. Subjects who did not receive a health checkup examination were excluded. Subjects with a history of prevalent AF were also excluded. We also excluded subjects with missing values in health checkup examination or questionnaire.

### Definition of covariates

Sociodemographic data included age, sex, and income. Low income was defined as having income in the lowest quartile among the entire Korean population. Comorbidities were included hypertension, diabetes, and dyslipidemia. Detailed definitions of comorbidities are presented in Supplementary Table S1. Body mass index (BMI) was defined as weight in kilograms divided by the square of height in meters (kg/m^2^). BMI as a categorical variable was defined as follow; underweight (< 18.5 kg/m^2^), normal weight (18.5 to < 23 kg/m^2^), overweight (23 to < 25 kg/m^2^), obese I (25 to < 30 kg/m^2^), and obese II (> 30 kg/m^2^)^27^. History of smoking status, alcohol consumption, and exercise were obtained with a health checkup self-reported questionnaire. Smoking status was classified as a non-smoker, ex-smoker, and current smoker. A pack-year (PY) was used to quantify the amount of smoking. Alcohol consumption was categorized as none, mild alcohol consumption (< 30 g/day), and heavy alcohol consumption (≥ 30 g/day). The questionnaire form and the assumptions for calculating the amount of alcohol consumption based on a questionnaire are described in the Supplementary Methods^15^. The frequency of alcohol intake per week (0 to 7 per week) and the amount of alcohol consumption per each drinking session (0 to ≤ 32 g, 32 to ≤ 56 g, 56 to ≤ 112 g, and > 112 g) were also collected^15^. From the self-reported questionnaire in the national health examination provided by Korean NHIC, the intensity and frequency of physical activity were obtained. The structured questionnaire was based on the International Physical Activity Questionnaire (IPAQ) which was developed by the World Health Organization^28,29^. Both the reliability and the validity of the Korean version of IPAQ short form were proven^30,31^. Light intensity of exercise was defined as ≥ 30 min per day of walking slowly or sweeping carpets. Moderate physical activity was defined as ≥ 30 min per day of brisk walking, dancing, or gardening, and strenuous physical activity was defined as ≥ 20 min per day of running fast, cycling, or aerobic^32^. The number of moderate or vigorous physical activities per week was also collected from the questionnaire. Regular exercise was defined as performing a moderate physical activity at least 5 times per week or strenuous physical activity at least 3 times per week^33^.

To assess the impact of clustering of unhealthy lifestyle factors, we divided the cohort into 8 groups by the prespecified combinations of unhealthy lifestyle habits, including current smoking, heavy alcohol consumption, and lack of regular exercise, as follows: (1) subjects without any unhealthy lifestyle factors; (2) those with a single unhealthy lifestyle factor among the following: current smoking, heavy alcohol consumption, or lack of regular exercise; (3) those with combinations of 2 unhealthy lifestyle factors, including current smoking with heavy alcohol consumption, lack of regular exercise with current smoking, and lack of regular exercise with heavy alcohol consumption; and (4) subjects with all 3 unhealthy lifestyle factors.

### Study outcomes

The study outcome of interest was new-onset AF. Newly diagnosed AF was identified when patients had the claims of relevant diagnostic codes (ICD-10-CM, I480-I484, and I489); either 1 diagnosis during hospitalization or at least 2 diagnoses in the outpatient clinic^3,34,35^. To assess the outcomes, the study population was followed up from the index health checkup and censored at the occurrence of outcome events, death, or at the end of the study period (December 31, 2017), whichever came first.

### Statistical analysis

Data are presented as number and percentage for categorical variables and mean ± standard deviation for continuous variables. The incidence rate of new-onset AF was calculated by dividing the number of events by the total follow-up period (per 1000 person-years). The association between unhealthy lifestyle factors and the incidence of AF was assessed using univariable and multivariable Cox proportional hazards regression. Unadjusted and adjusted hazard ratio (HR) and 95% confidence interval (CI) were analyzed. For multivariable analysis, sex, hypertension, diabetes, dyslipidemia, BMI, current smoking, alcohol consumption, regular exercise, and low income were included as adjusted variables. Since all study subjects were 66 years old, subjects’ age was not included in the model. The proportional hazards assumption for Cox models was graphically tested with a log minus log graph and verified with the Schoenfeld residuals.The assumption of proportionality was valid.

To assess the dose–response relationship between each unhealthy behavior and the risk of incident AF, we conducted the additional analyses. For smoking, the AF risk of ex-smoker < 10 PY, ex-smoker 10 to < 20 PY, ex-smoker ≥ 20 PY, current smoker < 10 PY, current smoker 10 to < 20 PY, and current smoker ≥ 20 PY were evaluated by multivariable Cox analysis compared to non-smoker group as a reference group. For alcohol consumption, the association between the frequency of alcohol consumption per week (once a week as a reference group) and the risk of AF was evaluated. The association between alcohol consumption among per each session (0 to ≤ 32 g as a reference group) and the risk of AF was also analyzed. For the exercise, the association between frequency of exercise per week in each level of exercise (light, moderate and strenuous physical activity) and the risk of AF was evaluated (4 times a week as a reference group.

A *p* value < 0.05 was considered statistically significant. We used SAS version 9.3 (SAS Institute, Cary, NC, USA) for statistical analyses.

## Results

**Figure 1:**
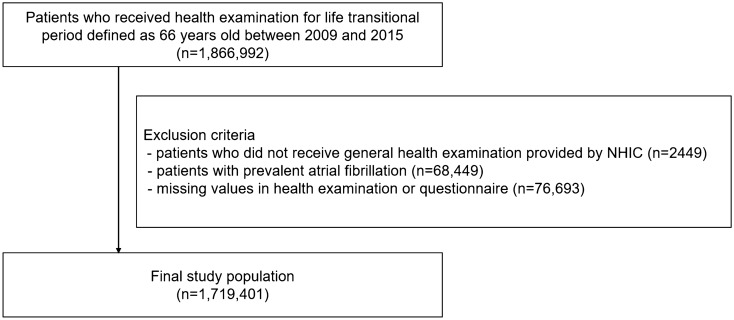
Study enrollment flow. *NHIC* national health insurance cooperation.

Figure 1 presents the study population enrollment flow. Finally, 1,719,401 subjects aged 66 years with baseline health checkup exam data were included in this analysis. During a 5-year mean follow-up period, 47,334 had incident AF (5.5 per 1000 person-years). Baseline characteristics are presented in Table 1. In this homogenous age group who were aged 66 years, the prevalence of current smokers was 12.6%, heavy alcohol consumption in 3.9%, and non-regular exercise in 52.6%. Patients with incident AF during follow-up had a higher prevalence of comorbidities such as hypertension and diabetes, but a lower prevalence of dyslipidemia (all *p* values < 0.001). Patients with incident AF showed higher mean BMI and waist circumference than those without AF (all *p* values < 0.001). Current smokers and subjects with heavy alcohol consumption were more common in patients with incident AF (all *p* values < 0.001). The proportion of subjects who performed regular exercise was higher in patients without AF during follow-up (*p* < 0.001).

**Table 1:** Baseline characteristics according to incident atrial fibrillation.

	Total (n = 1,719,401)	No AF (n = 1,672,067)	AF (n = 47,334)	*p* value
Male sex	791,084 (46.0)	765,184 (45.8)	25,900 (54.7)	< 0.001
**Comorbidities**				
Hypertension	911,899 (53.0)	882,207 (52.8)	29,692 (62.7)	< 0.001
Diabetes	351,803 (20.5)	340,858 (20.4)	10,945 (23.1)	< 0.001
Dyslipidemia	666,433 (38.8)	648,504 (38.8)	17,929 (37.9)	< 0.001
Body mass index (kg/m^2^)	24.3 ± 3.0	24.3 ± 3.0	24.7 ± 3.2	< 0.001
Waist circumference (cm)	83.1 ± 8.3	83.0 ± 8.3	84.8 ± 8.5	< 0.001
**Smoking**				
Non-smoker	1,193,180 (69.4)	1,162,942 (69.6)	30,238 (63.9)	< 0.001
Ex-smoker	309,492 (18.0)	299,831 (17.9)	9661 (20.4)	
Current smoker	216,729 (12.6)	209,294 (12.5)	7435 (15.7)	
**Alcohol consumption**				
Non	1,229,567 (71.5)	1,197,808 (71.6)	31,759 (67.7)	< 0.001
Mild (< 30 g/day)	422,056 (24.6)	409,172 (24.5)	12,884 (27.2)	
Heavy (≥ 30 g/day)	67,778 (3.9)	65,087 (3.9)	2691 (5.7)	
Regular exercise	815,298 (47.4)	793,949 (47.5)	21,349 (45.1)	< 0.001
Low income	496,405 (28.9)	482,328 (28.9)	14,077 (29.7)	< 0.001

*AF* atrial fibrillation.

### Factors associated with incident AF

In unadjusted univariable Cox proportional hazard model (Supplementary Table S2), hypertension, diabetes, dyslipidemia, being underweight, overweight, and obese and low income were associated with a higher incidence of AF. Female sex was associated with a lower risk of AF. In univariable analysis, both ex-smoker and current smokers were associated with an increased risk of AF compared to non-smoker (HR 1.32, 95% CI 1.29–1.35 for ex-smoker; HR 1.37, 95% CI 1.34–1.41 for the current smoker). Mild and heavy alcohol consumption also showed significant association with an increased risk of AF compared with subjects without alcohol consumption (HR 1.22, 95% CI 1.19–1.24 for mild alcohol consumption; HR 1.56, 95% CI 1.50–1.62 for heavy alcohol consumption). Subjects without regular exercise were associated with a modest increase of AF risk than those performing the regular exercise (HR 1.07, 95% CI 1.05–1.09).

### Lifestyle and risk of incident AF

Using a multivariable Cox proportional hazard regression model, hypertension, diabetes, being underweight, overweight, and obese, current smoking, heavy alcohol consumption, lack of regular exercise and low income were independently and significantly associated with an increased risk of AF (Supplementary Table S2). Without considering a combination of lifestyle risk factors, heavy alcohol consumption was associated with a 17% increase of AF risk compared to non-drinkers (HR 1.17, 95% CI 1.12–1.22). Lack of regular exercise and current smoking were associated with 11% and 10% increase of AF risk, respectively (HR 1.11, 95% CI 1.09–1.13 for lack of regular exercise; HR 1.10, 95% CI 1.07–1.13 for current smoking).

### Dose–response relationship between lifestyle risk factors and risk of AF

Based on the questionnaire, we evaluated the dose–response relationship between each lifestyle risk factors and the risk of AF.

**Figure 2:**
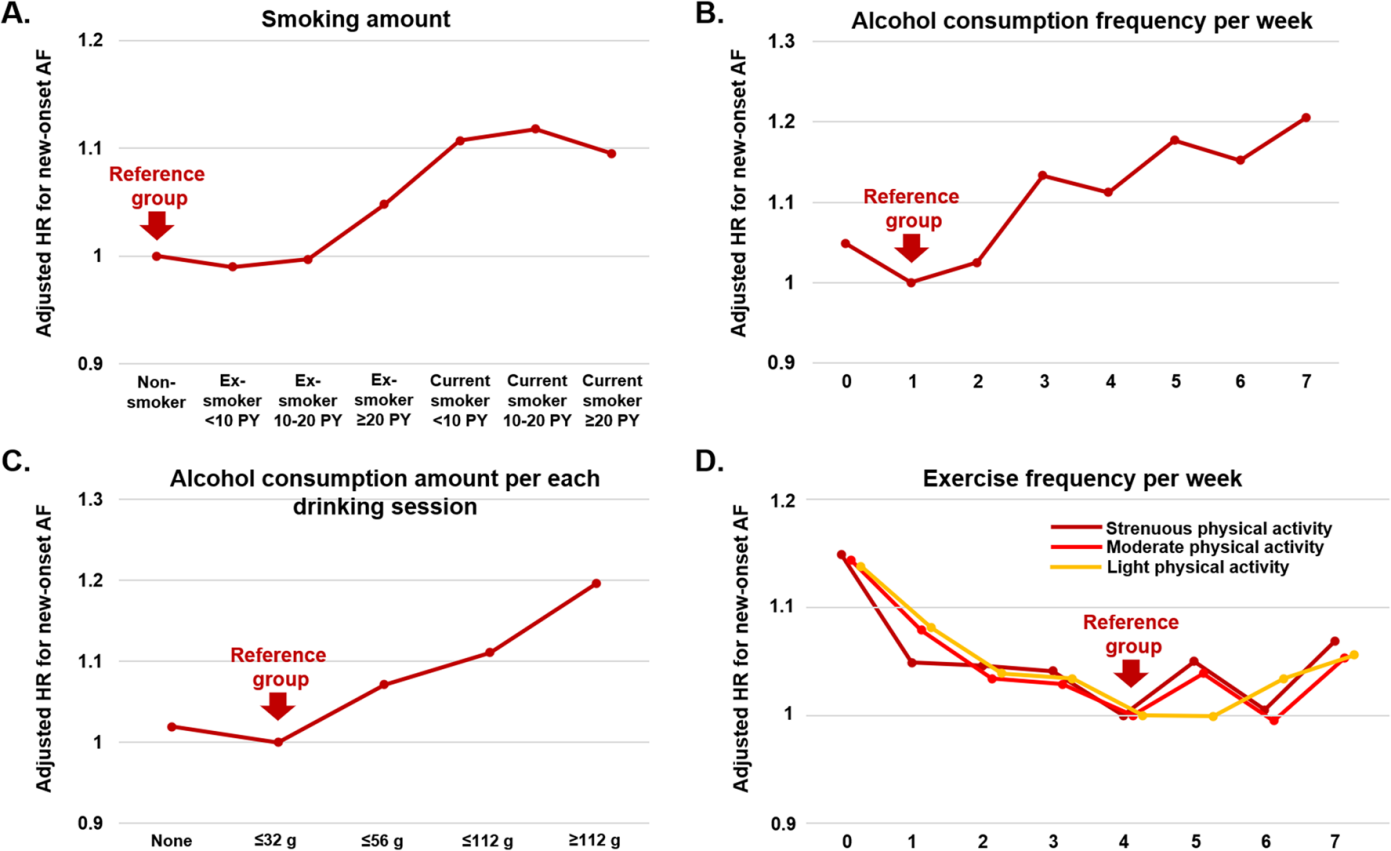
Dose–response relationship between each unhealthy lifestyle factor and risk for new-onset atrial fibrillation. (**A**) Smoking amount. (**B**) Alcohol consumption frequency per week. (**C**) Alcohol consumption amount per each drinking session. (**D**) Exercise frequency per week. *AF* atrial fibrillation, *HR* hazard ratio, *PY* pack-year.

Ex-smokers less than 10 pack-years and 10 to < 20 pack-years did not show a significant association with the risk of AF, compared to non-smokers. Even amongst ex-smokers, those with ≥ 20 pack-year ex-smokers were associated with a higher risk of AF (HR 1.05, 95% CI 1.01–1.08) (Fig. 2A and Supplementary Table S3). For current smokers, < 10 pack-year, 10 to < 20 pack-year and ≥ 20 pack-year showed a significant association with a higher risk of AF compared to non-smokers and HRs were similar in those 3 groups (Fig. 2A).

In comparison with subjects who drink once per week (reference group), subjects who drank ≥ 3 times in a week had a significantly higher risk, and those who drank every day had the highest risk of incident AF (HR 1.21, 95% CI 1.14–1.28) (Fig. 2B and Supplementary Table S3). The risk of AF was slightly higher in subjects who do not drink than those who drank once per week (HR 1.05, 1.02–1.08). There was a significant positive association between the amount of alcohol consumption per drinking session and the risk of AF. Subjects who drank ≤ 32 g per drinking session was used as a reference group. Non-drinkers did not show a significant association with a risk of AF. The risk of AF increased as the alcohol intake amount per drinking session increased (Fig. 2C and Supplementary Table S3). There was a 20% higher risk of AF in those who drank more than 112 g of alcohol per drinking session compared with the reference group.

Lack of regular exercise frequency is also associated with a higher risk of AF regardless of the intensity of exercise (Fig. 2D and Supplementary Table S3).

### Combinations of lifestyle risk factors and risk of AF

**Figure 3:**
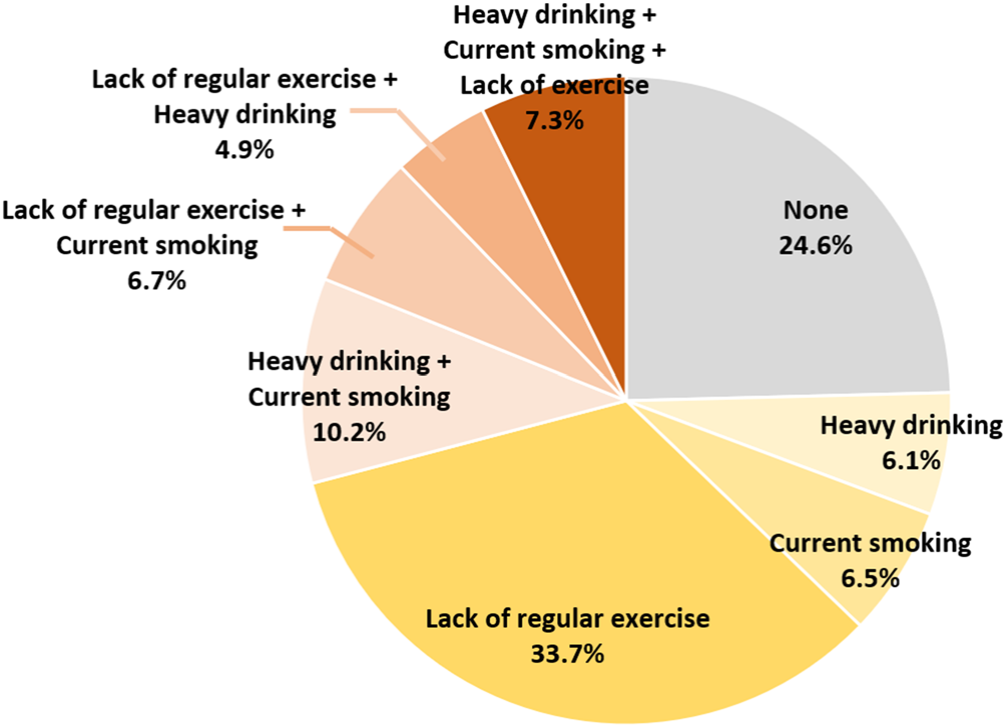
Distribution of unhealthy lifestyle factors.

Categorizing subjects with the combination of unhealthy lifestyle factors, 24.6% of subjects did not have any unhealthy lifestyle factors, 46.3% had only one unhealthy lifestyle risk factor, 21.8% had two unhealthy lifestyle risk factors, and 7.3% had three unhealthy lifestyle factors (Fig. 3).

**Figure 4:**
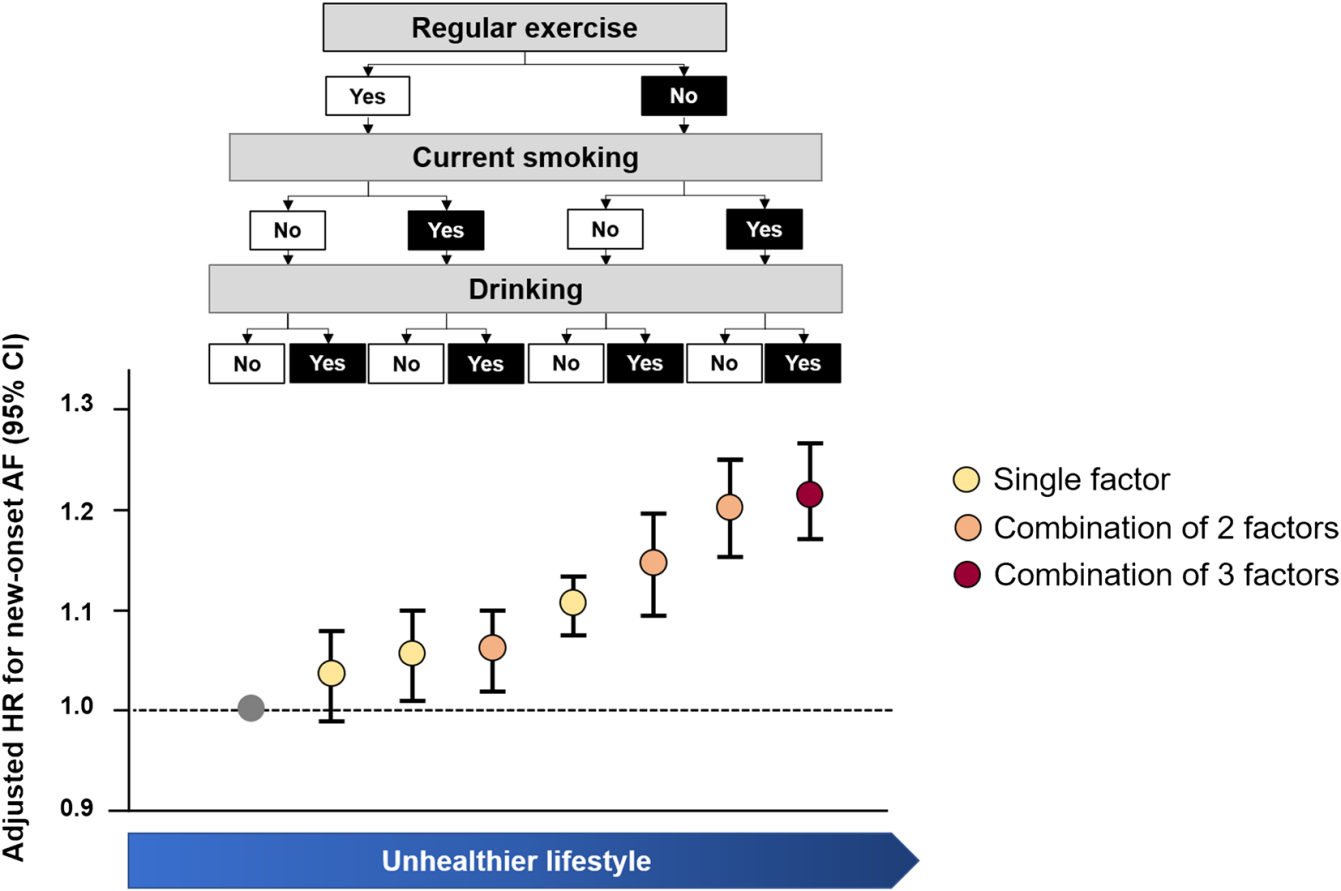
Combination of unhealthy lifestyle factors for risk of new-onset atrial fibrillation. *AF* atrial fibrillation.

As a single unhealthy lifestyle factor, lack of regular exercise was most common (33.1%), followed by current smoking (6.5%) and heavy alcohol consumption (6.1%). Using a multivariable Cox proportional hazard model, lack of regular exercise as a single unhealthy lifestyle factor was the strongest association with increased AF risk (HR 1.11, 95% CI 1.08–1.13) (Table 2 and Fig. 4). Subjects with current smoking as a single unhealthy lifestyle showed a modest higher risk for AF (HR 1.06, 95% CI 1.01–1.10). Subjects with heavy alcohol consumption as a single unhealthy lifestyle factor did not show a statistically significant higher risk for AF (HR 1.04, 95% CI 0.99–1.08).

**Table 2:** Unhealthy life habits and the incidence of atrial fibrillation.

Lack of exercise	Current smoking	Heavy drinking	Total number	Event number	IR^a^	Total population
						HR^b^ (95% CI)
No	No	No	423,580	9598	4.54	1 (reference)
		Yes	104,812	2990	5.73	1.04 (0.99–1.08)
	Yes	No	111,129	3304	6.13	1.06 (1.01–1.10)
		Yes	175,777	5457	6.40	1.06 (1.02–1.10)
Yes	No	No	580,261	14,985	5.04	1.11 (1.08–1.13)
		Yes	84,527	2665	6.26	1.15 (1.10–1.20)
	Yes	No	114,597	3872	6.91	1.21 (1.16–1.26)
		Yes	124,718	4463	7.30	1.22 (1.17–1.27)

*CI* confidence interval, *HR* hazard ratio, *IR* incidence rate.

^a^IR, per 1000 person-years.

^b^Adjusted for sex, hypertension, diabetes, dyslipidemia, body mass index, current smoking, alcohol consumption, regular exercise, low income.

Amongst combinations of two unhealthy lifestyle factors, current smoking with heavy alcohol consumption was most common (10.2%), followed by a lack of regular exercise with current smoking (6.7%) and lack of regular exercise with heavy alcohol consumption (4.9%). Among these combinations, current smoking with heavy alcohol consumption, lack of regular exercise with heavy alcohol consumption, and lack of regular exercise with current smoking were associated with a 6%, 15%, and 20% higher risk of incident AF, respectively. Subjects with the combinations of three unhealthy lifestyle factors, a cluster of ‘current smoking, heavy drinking, and lack of regular exercise’, was associated with a 22% higher risk of AF (HR 1.22, 95% CI 1.17–1.27).

## Discussion

In this large-scale homogenous age group cohort who were aged 66 years, our principal findings are as follows: (1) heavy alcohol consumption, lack of regular exercise, and current smoking were associated with 17%, 11%, and 10% higher risk for new-onset AF, respectively; (2) the clustering of unhealthy lifestyle factors was commonly observed, in about one-third of total population; and (3) the number of unhealthy lifestyle factors showed linear correlations with the risk of new-onset AF. As far as we are aware, this is the largest nationwide cohort assessing the impact of the combination(s) of unhealthy lifestyle risk factors on incident AF in population-based cohort of subjects aged 66 years. This study is also the first to investigate this in aspect in Asian population.

There are many known comorbidities and conditions which are independently associated with AF^19,21^. Among modifiable lifestyle factors, former or current smoking (HR 1.32 and 2.05, respectively), heavy alcohol consumption (risk ratio [RR] 1.14 for 15–21 drinks/week and 1.39 for > 21 drinks/week), and habitual vigorous exercise (RR 1.20 for 5–7 days/week) are emphasized in the guidelines^9,19–21^. Based on previous studies reporting the association between unhealthy lifestyle factors and incident AF in general population, leisure-time physical activity was associated with incident AF in a J-shaped pattern that meant moderate physical activity was associated with a reduced risk of incident AF (HR 0.81, 95% CI 0.68–0.87), whereas higher activity levels attenuated the benefits of moderate activity (HR 1.37, 95% CI 0.77–2.43)^12^. Although there remains some controversy, smoking is also associated with a modest increase of AF risk in a particular subset of the population^36,37^, being stronger in young and those with prior cardiovascular disease^36^.

Alcohol consumption is an important risk factor for incident AF through direct effects on the atrial substrate, and by contributing to other comorbidities such as hypertension, obesity, and obstructive sleep apnea^17^. Indeed, moderate drinking as well as binge drinking predispose to AF^17^. Moderate alcohol consumption has been associated with large left atrial scar burden^38^, and frequent drinking and the total amount of alcohol consumption per week were significant risk factors for incident AF, whereas the amount of alcohol consumption per each drinking session was not necessarily an independent risk factor^15^.

Our observations are largely consistent with previous results, whereby in this study population aged 66 years, each of heavy alcohol consumption, lack of regular exercise, and current smoking were independently associated with 17%, 11%, and 10% higher risks of AF. With a dose–response relationships, lifetime smoking amounts over 20 pack-year in ex-smokers and 10 pack-year in current smokers were significantly associated with a higher risk of AF compared to those with non-smokers. Also, both the frequency and the amount of alcohol consumption per each drinking session showed a positive correlation with a higher risk of AF. Regardless of the intensity of physical activity, low frequency of exercise less than 4 times per week was associated with a higher risk of AF and daily exercise slightly attenuated the benefits of moderate exercise frequency, consistent with previous studies^12,39^.

Unhealthy lifestyle risk factors are infrequently seen in isolation, and are commonly clustered in the same individual^22–24,40^. Approximately 20% of the general adult population have multiple lifestyle risk factors^22^. The synergy of these clustered unhealthy lifestyle factors has been reported for all-cause mortality^40^. In this study, subjects who had more unhealthy lifestyle risk factors (smoking, diet, physical activity, and abdominal obesity) showed higher all-cause mortality^40^. However, the evidence is limited regarding the relationship between the combination of unhealthy lifestyle factors including smoking, heavy alcohol consumption, and lack of regular exercise, and the risk of AF.

The focused study population of this study, ie. aged 66 years, poses relevant clinical implications. From age 65 onwards, for example, the CHA_2_DS_2_-VASc score becomes 1 in males and 2 in females, which requires consideration for stroke prevention^35,41^. Age factor is the most powerful factor for stroke risk prediction among the components of the CHA_2_DS_2_-VASc score^42,43^. Hence, finding potentially modifiable risk factors and preventing AF onset is important to reduce future complications associated with AF^4,43^. In this study, the clustering of unhealthy lifestyle factors was observed in approximately 30% of the total study population. Although combinations of unhealthy lifestyle factors were generally associated with a higher risk of AF, subjects with a combination of current smoking and heavy alcohol consumption did not show a statistical significance on the risk of AF compared to those who had a single factor as lack of regular exercise. The combination of 3 unhealthy lifestyle factors was associated with a 22% higher risk of new-onset AF. A comprehensive understanding of the impact of unhealthy lifestyle factors on the risk of AF and appropriate interventions to avoid these unhealthy habits is crucial to promote a healthy lifestyle and thus, prevent AF.

### Study limitations

This study had several limitations. First, the study design was a retrospective observational cohort. Therefore, the results of this study did not demonstrate causality, but shows associations. Second, although a randomized controlled prospective study could not be conducted using already known unhealthy lifestyle factors, residual confounding factors might exist. Third, the estimation of unhealthy behaviors including smoking, drinking, and lack of regular exercise was performed based on the self-reporting questionnaires administered to the health checkup participants, so recall bias is a possibility. Fourth, the questionnaire contained limited information about detailed lifestyle behaviors. For example, for exercise, only the information about aerobic exercise was included, and the quantification of smoking, alcohol consumption, and exercise was relatively crude. Lastly, changes in baseline lifestyle behavior during follow-up were not collected and adjusted for. However, the strength of this study is evaluating the impact of unhealthy lifestyle factors and its clustering on the risk for new-onset AF in a large-scale homogeneous age group from a nationwide population sample.

## Conclusions

Increased numbers of unhealthy lifestyle factors were associated with a higher risk of incident AF. These findings support the promotion of a healthy lifestyle to lower the risk of new-onset AF, including regular exercise, and avoiding smoking and heavy drinking.

## Supplementary information

Supplementary Information.
