# Supplementary material for: Association between clustering of unhealthy lifestyle factors and risk of new-onset atrial fibrillation: a nationwide population-based study
Source: Sci Rep. 2020 Nov 5;10:19224. doi: 10.1038/s41598-020-75822-y (PMC7645499; doi:10.1038/s41598-020-75822-y)
Supplement: Supplementary file 1 — Supplementary Information. [file 41598_2020_75822_MOESM1_ESM.docx]

**Title: Association between clustering of unhealthy lifestyle factors and risk of new-onset atrial fibrillation: a nationwide population-based study**

So-Ryoung Lee, MD,^1^ Eue-Keun Choi,^1,2^* MD, PhD, Hyo-Jeong Ahn, MD,^1^ Kyung-Do Han, PhD,^3^ Seil Oh, MD, PhD^1,2^, Gregory Y. H. Lip, MD^2,4,5^

^1^ Department of Internal Medicine, Seoul National University Hospital, Seoul, Republic of Korea

^2^ Department of Internal Medicine, Seoul National University College of Medicine, Seoul, Republic of Korea

^3^ Statistics and Actuarial Science, Soongsil University, Seoul, Republic of Korea

^4^ Liverpool Centre for Cardiovascular Science, University of Liverpool and Liverpool Chest & Heart Hospital, Liverpool, United Kingdom; and ^5^ Department of Clinical Medicine, Aalborg University, Aalborg, Denmark

**Supplementary Materials**

**Supplementary Methods**

**Supplementary Tables**

**Supplementary Methods**

*Questionnaire for alcohol consumption and assumption for calculating of the amount of alcohol consumption*

The questionnaire regarding alcohol consumption for the Korean nationwide health examination is as follows (translated into English).

| Please read the following questions and fill out your current situation.  On average, how many days a week do you drink?  □0 □1 □2 □3 □4 □5 □6 □7  How much do you usually drink a day when you drink (cup)? _______  (※ We calculate with glass used for each liquor type. One can of beer (355 cc) is equivalent to 1.6 cups of beer. |
| --- |

In Korea, a cup of beer contains 220 ml of beer, and a cup of Soju (Korean traditional alcohol beverage) contains 50 ml of Soju. We assumed that beer contains 4.5% of alcohol, a Soju contains 21.0% of alcohol, and the specific gravity of alcohol is 0.79. According to these assumption, a cup of beer contains 7.8 g of alcohol (220 * 0.045 * 0.79 = 7.821) and a cup of Soju contains 8.3 g of alcohol (50 * 0.21 * 0.79 = 8.295). Therefore, we assumed that a standard drink (one cup for each alcohol type) contains 8 g of alcohol. Although we calculated the amount of alcohol consumption based on beer and Soju because the majority of alcohol consumption in Korea was based on beer and Soju, in contrast to western countries according to the data from the Korean Customs Service and Korean Alcohol Liquor Industry Association (http://www.kalia.or.kr), other alcohol types such as whiskey and wine have their own glass which contains a similar amount of alcohol compared with our calculation based on beer and Soju.

**Supplementary Tables**

**Supplementary Table S1. Definition of comorbidities**

| **Disease** | **Definitions** |
| --- | --- |
| **Hypertension** | At least one diagnosis (I10-13 or I15) per year and at least one claim per year for the antihypertensive medication  Or systolic/diastolic blood pressure ≥ 140/90 mmHg |
| **Diabetes** | At least one diagnosis (E10-14) per year and at least one claim per year for the prescription of antidiabetic medication  Or fasting glucose level ≥ 126 mg/dL |
| **Dyslipidemia** | At least one diagnosis (E 78) per year and at least one claim per year for the prescription of a lipid-lowering agent  or Total cholesterol ≥ 240 mg/dL |

**Supplementary Table S2. Unadjusted and adjusted hazard ratios of demographic variables and comorbidities for the risk of atrial fibrillation**

|  | **Number**  **AF (Total)** | **IR*** | **Unadjusted HR**  **(95% CI)** | **Adjusted HR^†^**  **(95% CI)** |
| --- | --- | --- | --- | --- |
| **Sex** |  |  |  |  |
| **Male** | 25,900 (791,084) | 6.66 | 1 (reference) | 1 (reference) |
| **Female** | 21,434 (928,317) | 4.55 | 0.68 (0.667-0.69) | 0.688 (0.67-0.71) |
| **Hypertension** |  |  |  |  |
| **No** | 17,642 (807,502) | 4.37 | 1 (reference) | 1 (reference) |
| **Yes** | 29,692 (911,899) | 6.50 | 1.49 (1.46-1.52) | 1.42 (1.39-1.44) |
| **Diabetes** |  |  |  |  |
| **No** | 36,389 (1,367,598) | 5.29 | 1 (reference) | 1 (reference) |
| **Yes** | 10,945 (351,803) | 6.36 | 1.21 (1.18-1.24) | 1.07 (1.05-1.10) |
| **Dyslipidemia** |  |  |  |  |
| **No** | 29,405 (1,052,968) | 5.47 | 1 (reference) | 1 (reference) |
| **Yes** | 17,929 (666,433) | 5.56 | 1.03 (1.01-1.05) | 1.00 (0.98-1.02) |
| **Body mass index** |  |  |  |  |
| **<18.5 kg/m^2^** | 949 (36,450) | 5.37 | 1.10 (1.03-1.17) | 1.12 (1.04-1.19) |
| **18.5-22.9 kg/m^2^** | 13,286 (544,957) | 4.90 | 1 (reference) | 1 (reference) |
| **23-24.9 kg/m^2^** | 12,499 (478,936) | 5.20 | 1.06 ((1.04-1.09) | 1.03 (1.01-1.06) |
| **25-29.9 kg/m^2^** | 17,948 (593,119) | 6.02 | 1.23 (1.20-1.26) | 1.17 (1.14-1.19) |
| **≥30 kg/m^2^** | 2,631 (65,024) | 8.20 | 1.68 (1.61-1.75) | 1.61 (1.54-1.68) |
| **Smoking** |  |  |  |  |
| **Non-smoker** | 30,238 (1,193,180) | 5.01 | 1 (reference) | 1 (reference) |
| **Ex-smoker** | 9,661 (309,492) | 6.52 | 1.32 (1.29-1.35) | 1.02 (0.99-1.05) |
| **Current smoker** | 7,435 (216,729) | 6.86 | 1.37 (1.34-1.41) | 1.10 (1.07-1.13) |
| **Alcohol consumption** |  |  |  |  |
| **Non** | 31,759 (1,229,567) | 5.13 | 1 (reference) | 1 (reference) |
| **Mild (< 30g/day)** | 12,884 (422,056) | 6.21 | 1.22 (1.19-1.24) | 1.00 (0.97-1.02) |
| **Heavy (≥ 30g/day)** | 2,691 (67,778) | 7.98 | 1.56 (1.50-1.62) | 1.17 (1.12-1.22) |
| **Regular exercise** |  |  |  |  |
| **Yes** | 21,349 (815,298) | 5.30 | 1 (reference) | 1 (reference) |
| **No** | 25,985 (904,103) | 5.68 | 1.07 (1.05-1.09) | 1.11 (1.09-1.13) |
| **Low income** |  |  |  |  |
| **No** | 33,257 (1,222,996) | 5.41 | 1 (reference) | 1 (reference) |
| **Yes** | 14,077 (496,405) | 5.74 | 1.06 (1.04-1.09) | 1.03 (1.01-1.05) |

*IR, per 1000 person-years

^†^Adjusted for sex, hypertension, diabetes, dyslipidemia, body mass index, current smoking, alcohol consumption, regular exercise, low income

Abbreviations: CI, confidence interval; HR, hazard ratio; IR, incidence rate.

**Supplementary Table S3. Dose-response relationship in each unhealthy lifestyle behavior**

|  | **Adjusted HR^†^**  **(95% CI)** |
| --- | --- |
| **Smoking amount** |  |
| **Non-smoker** | 1 (reference) |
| **Ex-smoker <10 PY** | 0.99 (0.95-1.04) |
| **Ex-smoker 10 to <20 PY** | 1.00 (0.95-1.04) |
| **Ex-smoker ≥20 PY** | 1.05 (1.01-1.08) |
| **Current smoker <10 PY** | 1.11 (1.04-1.18) |
| **Current smoker 10 to <20 PY** | 1.12 (1.06-1.18) |
| **Current smoker ≥20 PY** | 1.10 (1.06-1.13) |
|  | |
| **Alcohol consumption frequency per week** | |
| **0** | 1.05 (1.02-1.08) |
| **1** | 1 (reference) |
| **2** | 1.03 (0.98-1.07) |
| **3** | 1.13 (1.08-1.19) |
| **4** | 1.11 (1.04-1.19) |
| **5** | 1.18 (1.10-1.26) |
| **6** | 1.15 (1.06-1.25) |
| **7** | 1.21 (1.14-1.28) |
| **Alcohol consumption amount per each session** | |
| **None** | 1.02 (0.99-1.05) |
| **≤32 g** | 1 (reference) |
| **≤56 g** | 1.07 (1.04-1.1) |
| **≤112 g** | 1.11 (1.06-1.17) |
| **≥112 g** | 1.2 (1.08-1.33) |
|  |  |
| **Frequency of exercise per week** |  |
| **Strenuous physical activity** |  |
| **0** | 1.15 (1.09-1.21) |
| **1** | 1.05 (0.99-1.12) |
| **2** | 1.05 (0.98-1.12) |
| **3** | 1.04 (0.98-1.11) |
| **4** | 1 (reference) |
| **5** | 1.05 (0.98-1.13) |
| **6** | 1.01 (0.92-1.09) |
| **7** | 1.07 (1.00-1.15) |
| **Moderate physical activity** |  |
| **0** | 1.14 (1.09-1.20) |
| **1** | 1.08 (1.02-1.14) |
| **2** | 1.03 (0.98-1.10) |
| **3** | 1.03 (0.97-1.09) |
| **4** | 1 (reference) |
| **5** | 1.04 (0.98-1.11) |
| **6** | 1.00 (0.93-1.07) |
| **7** | 1.05 (0.99-1.12) |
| **Walking** |  |
| **0** | 1.14 (1.10-1.18) |
| **1** | 1.08 (1.03-1.14) |
| **2** | 1.04 (0.99-1.09) |
| **3** | 1.03 (0.99-1.08) |
| **4** | 1 (reference) |
| **5** | 1.00 (0.95-1.04) |
| **6** | 1.03 (0.99-1.09) |
| **7** | 1.06 (1.01-1.10) |

^†^Adjusted for sex, hypertension, diabetes, dyslipidemia, body mass index, current smoking, alcohol consumption, regular exercise, low income

Abbreviations: CI, confidence interval; HR, hazard ratio; IR, incidence rate.
